# Resource Optimization for Multi-Unmanned Aerial Vehicle Formation Communication Based on an Improved Deep Q-Network

**DOI:** 10.3390/s23052667

**Published:** 2023-02-28

**Authors:** Jie Li, Sai Li, Chenyan Xue

**Affiliations:** 1 College of Electronic and Information Engineering, Nanjing University of Aeronautics and Astronautics, Nanjing 211106, China; 2 Leihua Electronic Technology Research Institute, Aviation Industry Corporation of China, Wuxi 214063, China

**Keywords:** UAV, U2B, U2U, DQN, CBAM, VDN

## Abstract

With the widespread application of unmanned aerial vehicle (UAV) formation technology, it is very important to maintain good communication quality with the limited power and spectrum resources that are available. To maximize the transmission rate and increase the successful data transfer probability simultaneously, the convolutional block attention module (CBAM) and value decomposition network (VDN) algorithm were introduced on the basis of a deep Q-network (DQN) for a UAV formation communication system. To make full use of the frequency, this manuscript considers both the UAV-to-base station (U2B) and the UAV-to-UAV (U2U) links, and the U2B links can be reused by the U2U communication links. In the DQN, the U2U links, which are treated as agents, can interact with the system and they intelligently learn how to choose the best power and spectrum. The CBAM affects the training results along both the channel and spatial aspects. Moreover, the VDN algorithm was introduced to solve the problem of partial observation in one UAV using distributed execution by decomposing the team q-function into agent-wise q-functions through the VDN. The experimental results showed that the improvement in data transfer rate and the successful data transfer probability was obvious.

## 1. Introduction

As a new technology, unmanned aerial vehicle (UAV) technology has been widely used in civil, public and military fields [1]. The effectiveness and potential of UAVs for coastal-zone applications were elaborated in a review [2]. The execution latency has been decreased and the computation performance has been improved for the mobile edge computing networks by integrating UAVs into the research [3]. By poring over a large amount of literature, Ref. [4] provided a novel insight into cyber physical systems in UAV networks. To maximize the quality of the experience of real-time video streaming, the authors of [5] designed the power control, the movement and the video resolution of the UAV to base station and UAV user links. Using the Stackelberg dynamic game, Ref. [6] came up with a resource pricing and trading scheme to realize edge computing resource allocation between UAVs and edge computing stations. Despite only one single UAV being able to finish some difficult tasks, the possibility of mission failure was relatively high, as tasks were getting more complex and diversified. Multi-UAV applications have attracted widespread attention and achieved remarkable accomplishments in some situations where one UAV may not suffice. To make full use of the advantages of the UAV, multi-UAV formations have been designed and widely used in many fields. The UAV formation technology has become an important research topic in the world because it is highly three-dimensional, informationalized and electronized. The UAV formation technology is mainly applied in data fusion, physical verification platform technologies, formation control, task assignment, flight path planning, communication networking and virtual and information perception. For a multi-UAV formation, multisource information fusion and information perception are the most important applications. The routing problem of multiple UAVs to realize remote sensing and area coverage in the shortest time was studied in [7]. To respond quickly to a disaster scenario, the authors of [8] evaluated open source and commercial software solutions for 3D reconstructions of disaster scenarios. The authors of Ref. [9] constructed a mobile sensing system based on multi-UAVs, which captured the variance of the air quality index at the meter level and plotted its corresponding fine-grained distribution. The authors of Ref. [10] solved the problem of cooperative control based on multi-UAV systems. Based on a multi-UAV wireless cache network, a new joint communication scheduling and trajectory method was proposed in [11]. To enhance the throughput of the network and minimize the data transmission time for a multi-hop UAV relay network, Ref. [12] addressed the packet routing problem. The authors of Ref. [13] solved the topology control problem for a UAV swarm network. Compared to other wireless networks, the topology of multi-UAV formation networks will always change as the positions of nodes, the amount of nodes and links are not fixed. The changes in position and speed can lead to intermittent connections between each one [1]. Both [14] and [15] agreed that the improvement of communication rate and the assurance of communication quality were necessary on account of the rapid different complexities and changing task surroundings. Reasonable resource allocation is an effective method to achieve these goals.

To address the problem of resource allocation, the random method is one of the traditional methods and has recently been put into use in joint power and spectrum resource distribution by a number of researchers. In general, the random method serves as a baseline for comparison. For instance, powers are evenly assigned to every transmitter and the subchannels are distributed randomly to the devices in the literature [16]. In addition to the random method, in the work of [17], Wentao Zhao et al. randomly assigned channels to every device to device pair, and then chose one power from the candidate power list that could maximize the data transfer rate of the system. The random method is superior in working speed but inferior in improving the communication capacity and transmission quality of the system. A resource optimization method combining spectrum and speed is proposed in reference [18]. In order to meet the requirements of high average spectral efficiency and to maximize the minimum average energy, the authors of [19] worked on optimizing the transmitted power and trajectory of UAVs and the power splitting ratio of the ground terminals.

Deep learning (DL) has been widely used in areas such as natural language processing and computer vision [20], as well as machine translation and automatic speech recognition [21]. The progress of reinforcement learning (RL) and deep convolutional neural networks (CNN) enabled machine learning (ML) to acquire unprecedented results in visual areas, for instance playing Atari games [22], image classification [23] and face recognition [24]. For the convolutional layer of one CNN, a single neuron is related to only a few adjacent neurons. One convolutional layer usually represents a few feature maps, and every feature map consists of certain rectangular neurons. The authors of [25] proved that some suitable learning mechanisms can help capture correlations and improve the performance of a CNN without additional oversight. The combination of deep learning and reinforcement learning is also known as deep reinforcement learning (DRL). Recently, DRL has also been put into use in the resource assignment problem of wireless networks. Compared to the traditional methods, DRL has its own superiority [26]. Dealing with large systems, a deep Q-network (DQN) is used to find a channel access strategy in the study [27]. A good strategy can be found directly from historical observations without knowing prior system information. Contrary to the above work, and to ensure the quality of communication, this manuscript considers the joint power and spectrum optimization of a UAV formation flight to maximize the transmission rate and increase the successful data transfer probability based on a DQN. The main contributions of this paper are as follows:

1. Resource optimization based on the three-dimensional distribution of multiple UAVs, which is different from a random [28] or flat distribution [29] of UAVs, as used in the previous study. The multi-UAVs were located successively at edges or the eight vertices of a cube and kept a cube formation during their flight;
2. In order to achieve joint power and channel resource optimization for the UAV formation, a DQN was used. Moreover, a CNN was used after the state data were preprocessed in the training process;
3. Based on a DQN, the convolutional block attention module (CBAM) and value decomposition network (VDN) modules were introduced to further realize system performance improvement. The CBAM worked at the CNN layer to capture correlations of the feature map along both the channel and spatial aspects, and the VDN worked as the reward mechanism. The experimental results proved the effectiveness of the introduction of the CBAM and VDN.

## 2. System Model

In this chapter, the scenario of a multi-UAV formation network and the data transmission model are introduced, respectively. In the channel model, both the small-scale and large-scale fading is included and the optimization objective function is given last.

### 2.1. Scenario Setting

It was assumed that the multi-UAVs kept the flying formation during the execution of a task. The whole UAV network consisted of multi-UAVs and one base station (BS). The multi-UAVs were located at the eight vertices or edges of one cube. There were two data transmission modes, and the spectrum availability was limited. For the UAV network, *M* (M=1,2,⋯,M) orthogonal uplinks were pre-assigned to M UAVs to perform the UAV to base station (U2B) data transfer, and *N* (N=1,2,3,⋯,N) pairs of UAV to UAV (U2U) links performed the U2U data transfer. The U2B links could be reused by the U2U links to make full use of the frequency. In one time slot, as agreed, one U2B link only used one uplink and multiple U2U links could reuse the same uplink; however, one U2U link could only occupy one uplink. The value $\rho_{n,m}$ was used as the n∗m binary indicator matrix and $\rho_{n,m}=1$ if the *n*th U2U link reused the *m*th uplink resource, otherwise $\rho_{n,m}=0$. $\left(x_{k},y_{k},z_{k}\right)$, $x_{j},y_{j},z_{j}$ and (0,0,H) were used to represent the locations of the *k*th UAV, the *j*th UAV and the BS, respectively. Then, the distance between the *k*th and the *j*th UAV is calculated as follows:

(1) $$d_{k,j}=\sqrt{{\left(x_{k}-x_{j}\right)}^{2}+{\left(y_{k}-x_{j}\right)}^{2}+{\left(z_{k}-z_{j}\right)}^{2}}.$$

Similarly, the distance between the *k*th UAV and the BS is calculated as

(2) $$d_{k,BS}=\sqrt{{\left(x_{k}-0\right)}^{2}+{\left(y_{k}-0\right)}^{2}+{\left(z_{k}-H\right)}^{2}}=\sqrt{x_{k}^{2}+y_{k}^{2}+{\left(z_{k}-H\right)}^{2}}.$$

### 2.2. Modeling the Line of Sight Probability

In this part, the channel model used in the multi-UAV formation network is introduced. The channel model consists of the channel model of the U2U and U2B data transfer. The U2U and U2B links are different due to the different line-of-sight (LoS) characteristics and the elevation angle in the actual environment.

For the U2U channel model, the free space channel model of the U2U link was used in the multi-UAV formation network. The values $P_{ntr}$ and $P_{nre}$ denote the transmitter and receiver power of the *n*th U2U link reusing the *m*th uplink. The relationship between them can be expressed as

(3) $$P_{nre}\left(t\right)=P_{ntr}h{\left(d_{ntr,nre}\left(t\right)\right)}^{-\omega },$$

where *h* is the constant channel gains factor related to the amplifier and antenna, $d_{ntr,nre}$ is the distance between the transmitter and receiver UAVs of the *n*th U2U link and ω is the channel path loss constant. The receiver interference of the *n*th U2U link consists of the data that comes from the *n*th U2B link and other U2U links that reuse the same *m*th uplink. $ThevalueP_{m}^{U2B}$ is labeled as the transmitter power of the *m*th U2B link, and, then, the receiver interference of the *n*th U2U link $I_{nre}$ is expressed as follows.

(4) $$I_{nre}\left(t\right)=P_{m}^{U2B}h{\left(d_{ntr,nre}\left(t\right)\right)}^{-\omega }+\sum_{n^{\prime }=1,n^{\prime }\neq n}^{N}\rho_{n^{\prime },m}P_{n^{\prime }}^{U2U}h{\left(d_{ntr,nre}\left(t\right)\right)}^{-\omega }+\sigma^{2},$$

where $P_{n^{\prime }}^{U2U}$ is labeled as the transmitter power of the ${n^{\prime }}^{th}$ U2U link, $\rho_{n^{\prime },m}$ indicates whether the ${n^{\prime }}^{th}$ U2U link reuses the *m*th uplink and $\sigma^{2}$ is the variance of the additive white Gaussian noise (AWGN) with a mean equal to zero. The data transfer rate of the U2U link can be calculated as follows.

(5) $$R_{n}^{U2U}\left(t\right)=log_{2}\left(1+\frac{P_{nre}\left(t\right)}{I_{nre}\left(t\right)}\right).$$

For the U2B channel model, the model of air-to-ground (ATG) propagation adopts the U2B data transmission proposed in the existing literature [30,31]. To predicte the ATG path loss, [32] designed a statistical propagation model. In time slot *t*, the path loss of LoS and non-line-of-sight (NLoS) of the *k*th U2U link is indicated as

(6) $$\begin{array}{c} PL_{LoS,k}\left(t\right)=L_{FS,k}\left(t\right)+20log\left(d_{k,BS(t)}\right)+\eta LoS,\\ PL_{NLoS,k}\left(t\right)=L_{FS,k}\left(t\right)+20log\left(d_{k,BS(t)}\right)+\eta NLoS, \end{array}$$

where $d_{k,BS}$ is the distance between the BS and transmitter UAV of the $k^{th}$ U2U link, the values ηLoS and ηNLoS are attenuation factors that are caused by the LoS and NLoS transmission, respectively. The value $L_{FS,k}\left(t\right)$ is the free space path loss that is expressed as follows.

(7) $$\begin{array}{c} L_{FS,k}\left(t\right)=20log\left(f\right)+20log\left(\frac{4\pi }{c}\right), \end{array}$$

where *f* is the carrier frequency. Recommended by the International Telecommunication Union (ITU), as mentioned in the reference [33], the probability of the LoS link data transfer is expressed as

(8) $$\begin{array}{c} P_{LoS,k}\left(t\right)=\frac{1}{1+\alpha exp(-\beta \left(\theta_{k}\left(t\right)-\alpha \right))}, \end{array}$$

where α and β are constants that are related to the environment and $\theta_{k}\left(t\right)$ is the elevation angle of the *k*th UAV. The sight probability model is adapted to the low-altitude aerial platforms of the UAV network. This probability is closely related to three statistical parameters in the environment: the average number of buildings per unit area; the proportion of construction land area in the total land area; the building height distribution based on the Rayleigh probability density function. The average path loss in decibels (dB) is expressed as follows.

(9) $$\begin{array}{c} PL_{avg,k}\left(t\right)=P_{Los,k}\left(t\right)*PL_{LoS,k}\left(t\right)+P_{NLos,k}\left(t\right)*PL_{NLoS,k}\left(t\right)\\ =P_{LoS,k}\left(t\right)*PL_{LoS,k}\left(t\right)+P_{NLoS,k}\left(t\right)*\left(1-PL_{LoS,k}\left(t\right)\right). \end{array}$$

The average power received by the BS from the *m*th U2B link is denoted as

(10) $$\begin{array}{c} P_{m,BS}\left(t\right)=\frac{P_{m}^{U2B}}{10^{PL_{avg,k}\left(t\right)/10}}, \end{array}$$

where $P_{m}^{U2B}$ is the transmitter power of the *m*th U2B link. Similarly, $P_{n}^{U2U}$ is labeled as the transmitter power of the *n*th U2U link that reuses the *m*th uplink. The interference received by the BS from the U2U links is calculated as

(11) $$\begin{array}{c} I_{m,BS}\left(t\right)=\sum_{n=1}^{N}\rho_{n,m}P_{n,BS}^{m}\left(t\right)=\sum_{n=1}^{N}\rho_{n,m}\frac{P_{n}^{U2U}}{10^{PL_{avg,n}\left(t\right)/10}}. \end{array}$$

Therefore, the signal-to-interference-plus-noise-ratio (SINR) of the *m*th U2B link is calculated as

(12) $$\begin{array}{c} \gamma_{m,BS}\left(t\right)=\frac{P_{k,BS}^{m}\left(t\right)}{\sigma^{2}+I_{m,U2U}\left(t\right)}, \end{array}$$

where $\sigma^{2}$ is the variance of the AWGN with a mean equal to zero. The data transfer rate of the *m*th U2B link is expressed as

(13) $$R_{m}^{U2B}\left(t\right)=log_{2}\left(1+\gamma_{m,BS}\left(t\right)\right).$$

### 2.3. The Problem Optimization Objective

This section details the optimization objective of the multi-UAV formation network. The goal of this manuscript was to improve the successful data transfer probability of the U2U links and realize the maximization of the system data transfer rate. The optimization objective is modeled as

(14) $$\begin{array}{cc} maxR_{system}= & max\sum_{T}\left(\sum_{m=1}^{M}R_{m}^{U2B}\left(t\right)+\sum_{n=1}^{N}R_{n}^{U2U}\left(t\right)\right)\\ s.t.\;\;\;\; & c1:P_{m}=P_{max}\\ \; & c2:P_{ntr}\leq P_{max}\\ \; & c3:\rho_{n,m}\in \left\{0,1\right\}\;\;\;\forall n\in N,m\in M, \end{array}$$

where $P_{max}$ stands for the maximum transmitter power used by the UAVs, $P_{ntr}$ and $P_{m}$ stand for the transmitter power of the *n*th U2U and *m*th U2B links, respectively. In view of the fact that the distances from the UAVs to the BS are longer than those between each UAV, the maximum power is adopted by the U2B link transmitters to guarantee the quality of the data transfer. For the sake of maximizing the data transfer rate and generating less interference to the system, each U2U link ought to choose the appropriate power and spectrum. Three power levels were set to be used by the U2U links. Then, $P_{ntr}$ and $\rho_{n,m}$ are the arguments to be optimized. The optimization function consists of the rate of U2U links, U2B links and the transmission time limit. The optimization function at each time slot *t* that was used as the reward function in the proposed DQN mechanism is expressed as follows.

(15) $$\begin{array}{cc} r_{t} & =a\sum_{m=1}^{M}R_{m}^{U2B}+b\sum_{n=1}^{N}R_{n}^{U2U}-\left(1-a-b\right)\left(T-E_{t}\right)\\ \; & =R_{t}+T_{t}, \end{array}$$

where *a*,*b* and (1−a−b) are the percentage of each part. If the data in the U2U links are transmitted successfully within the transmission time *T*s, it is classed as a successful data transfer. The value $E_{t}$ is the remaining time left to finish the data transfer in the U2U links and it is initialized to *T*. The third section is initialized to zero, which means the transmitters have a lot of time to transmit data to obtain a bigger reward function $r_{t}$. If the remaining time $E_{t}$ gets shorter, the reward function $r_{t}$ can reduce due to the increase in the failure data transfer probability.

## 3. The Proposed Method

This section details the joint spectrum and power allocation method based on a DQN in the multi-UAV formation network. The process of the DQN method is introduced first, and, then the algorithm of the CBAM and the VDN module is added, based on the DQN.

### 3.1. Introduction of the DQN

The multi-UAV movement problem could be modeled as the constrained Markov decision process (CMDP) problem, giving a moving area that is classical in order to solve RL tasks with constraints. The maximum cumulative long-term discounted reward can be obtained after the agents are well trained to execute the best action strategy. In the multi-UAV formation network, the whole network corresponds to the environment in the RL method, and each U2U link is regarded as an agent. An action is labelled with $a_{t}$ and it includes two parameters of the action space *A*. Each U2U transmitter makes an action choice according to the current state $s_{t}$, and the environment feedbacks a reward $r_{t}$ and the next state $s_{t+1}$. Therefore, state, action, reward and the next state $\left[s_{t},a_{t},r_{t},s_{t+1}\right]$ are the main transitions in RL. The value *A* is a set of *M* uplinks and three power levels. The state $s_{t}$ contains the channel information in the U2B links $H_{UB}$ and U2U links $H_{uu}$, the interference from the U2U links $I_{uu}$, the uplink reuse indicator of the U2U links *B*, the time left to transmit data $E_{t}$ and the data left to transmit $D_{t}$. The set of all states is called the state space *S*.

For a given state $s_{t}$, in each time slot the U2U link transmitter needs to select an action according to the strategy π. In regular Q-learning, the decision-making strategy is carried out by a q-function. Once a state–action pair is determined, the q-value reward is calculated after selecting one action. To find the optimization strategy to maximize the cumulative reward, once the q-value is calculated, the action selection strategy can be carried out according to the following expression

(16) $$\begin{array}{c} a_{t}=max_{a\in A}Q\left(s_{t},a\right). \end{array}$$

So, the optimization strategy can be denoted as $\pi :s_{t}\in S\to a_{t}\in A.$

For a specific action and state, the maximum Q-value, i.e, the expected accumulative discount reward, is expressed as

(17) $$Q\left(s,a\right)=E\left[\sum_{i=0}^{\infty }\lambda^{i}r_{t+i}\right],$$

where λ is the discount coefficient that is needed to strike a balance between current and future decisions. Equation (17) is the total cost for the optimization. According to Equation (15), each UAV can choose the most appropriate power and spectrum based on its remaining data transmission time and the system interference.

In the multi-UAV formation problem, the state and action space will be very large, which makes it is difficult to use regular Q-learning as some Q-values are seldom updated. As a combination of RL and a deep network, the DQN has been widely used. A DQN can take a state as the input and output all the action values Q(s,a;θ). It can then select the action with the maximum value as the next action. The values of θ are the parameters of the neural network (NN) and are called the Q-network. As is well-known, in the study of [22], Mnih et al. introduced target networks and experience replay to achieve a better performance for the DQN algorithm. The output of target network in the DQN is written as

(18) $$\begin{array}{cc} y_{t}^{DQN} & =r_{t+1}+\gamma max_{a}Q\left(s_{t+1},a;\hat{\theta }\right)\\ \; & =r_{t+1}+\gamma max_{a}E\left[\sum_{i=1}^{\infty }\lambda^{i}\left(R_{t+i}+T_{t+i}\right)\right], \end{array}$$

where $\hat{\theta }$ are the coefficients of the target network and are updated according to the Q-network after certain steps. The coefficients of the Q-network are updated according to the gradient descent policy to loss function. The loss function is shown as below.

(19) $$\begin{array}{c} Loss\left(\theta \right)=\sum_{\left(s_{t},a_{t}\in D\right)}{\left(y-Q\left(s_{t},a_{t};\theta \right)\right)}^{2} \end{array}$$

where *D* is a memory buffer that is used to store all the transitions $\left[s_{t},a_{t},r_{t},s_{t+1}\right]$. Referring to the experience replay method, in order to reduce the data correlation, fixed batch transitions are randomly selected from D in every training period. As the input of the Q-network, parameters of the Q-network are updated while training the data.

### 3.2. The DQN with the CBAM

Research [22] has proven that the introduction of the experience replay and target network has greatly improved the performance of the DQN. In this system, the state data are converted into a square matrix to train the CNN. On this basis of the DQN, the CBAM is added, which consists of two important submodules: channel and spatial. The channel submodule adopts both average-pooling and max-pooling outputs, processed by a shared network. The outputs are then processed by the spatial submodule. In the spatial submodule, passing through the pooling layer, the output data of the channel submodule are put into a convolution layer. The following details the operation of the submodules.

Let $F_{avg}^{c}$ and $F_{max}^{c}$ represent the average-pool and max-pool operations, respectively. Two different descriptors are generated after the pooling operation, acting on the UAV states. The channel attention data $M_{c}\in R^{C*1*1}$ can be obtained after the pooling results are both processed by the following shared network. The shared network consists of a multi-layer perceptron (MLP) that has one hidden layer, and the size of the hidden activation is $R^{C/r*1*1}$, where *r* is the reduction ratio. The output feature data are summed element-wise after the shared network. So, the channel attention operation can be expressed  as

(20) $$\begin{array}{cc} M_{c}\left(F\right) & =\sigma (MLP(AvgPool(F))+MLP(MaxPool(F)))\\ \; & =\sigma (W_{1}\left(W_{0}\left(F_{avg}^{c}\right)\right)+W_{1}\left(W_{0}\left(F_{max}^{c}\right)\right)), \end{array}$$

where σ represents the sigmoid function, $W_{0}$ and $W_{1}$ are the weights of the shared network and they are shared for both inputs’ pooling results, $W_{0}\in R^{C/r*r}$ and $W_{1}\in R^{C*C/r}$.

The spatial attention graph is generated by using the spatial relationships of the features that focus on ‘where’ the useful information is. The spatial attention is supplement to the channel attention. For the spatial attention, the average-pooling and max-pooling operations are applied first to concatenate the channel attention outputs to a resultful characteristic descriptor. Then, the descriptor is processed by a convolution layer to obtain the spatial attention data $M_{s}\left(F\right)\in R^{HW}$. So, the spatial attention operation can be expressed as

(21) $$\begin{array}{cc} M_{s}\left(F\right) & =\xi (f([AvgPool(F);MaxPool(F)]))\\ \; & =\xi (f\left(\left[F_{avg}^{s};F_{max}^{s}\right]\right)), \end{array}$$

where ξ represents the sigmoid function and *f* denotes a convolution operation.

### 3.3. The DQN with the VDN

Section 3.2 details the optimization of the network from the channel and spatial aspects, and this section looks at improving the system performance in terms of the reward mechanism. Due to partial observability, the spurious rewards problem exists in both the fully centralized and decentralized mechanisms. To avoid the spurious reward data produced by the naive independent agents, a VDN is introduced while training the individual agents. The operation is to obtain agent-wise value functions by decomposing the team value function. Based on team rewards, the system can acquire optimal linear decomposition learning. The total Q-gradient is propagated backward by the deep neural network (DNN) representing the function of each agent-wise value.

For the multi-UAV formation network, distributed across *n* UAVs, the observations and actions are labeled as n-dimensional tuples of observations in *O* and actions in *A*.

**Figure 1:**
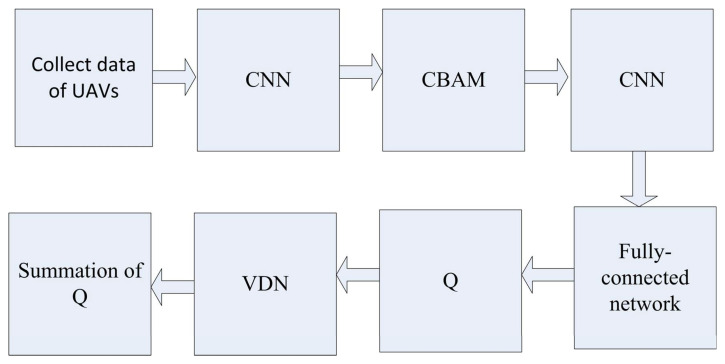
The overall improved DQN architecture with the CBAM and VDN.

The additive decomposition can be expressed by the following equation.

(22) $$\begin{array}{c} Q\left(\left(h^{1},h^{2},\cdots ,h^{n}\right),\left(a^{1},a^{2},\cdots ,a^{n}\right)\right)\approx \sum_{i=1}^{n}\tilde{Q_{i}}\left(h^{i},a^{i}\right), \end{array}$$

where the $\tilde{Q_{i}}$ represent every agent’s own observations and is learned by back propagating gradients according to the joint summation reward, rather than the specific reward of agent *i*, $\left(h^{1};h^{2};\cdots ,h^{n}\right)$ is the history tuple of one agent. For the improved summation reward, $\tilde{Q_{i}}$ are action–value functions that consist of all particular rewards of the UAVs, without any other constraints. The overall architecture is shown in Figure 1.

### 3.4. Training and Validation

Algorithm 1 is the flow of the entire training architecture. The buffer capacity *D* is $10^{6}$, the training step *M* is 40000 and every 2000 steps represents one episode. The value γ is 0.99 and *K* is 50. The value λ of the Q-function is 1. The programming language is python and it was written and executed on PyCharm Community Edition 2019.2.2 x64 using Xeon Silver 4110 CPU @ 2.10 GHz @ 2.10 GHz. The network layer is detailed in the simulation section. The communication rate and time delay were designed in the Q-function, in order to maximize the Q-function and obtain a better training effect, and the CBAM and VDN were added, respectively. The algorithm is based on a pure DQN without 1 and 2 modules. Any module can be embedded into the network quickly. The CBAM affects the training results along both channel and spatial aspects. With the VDN, the action selection for the UAVs is based on the global observations of all UAVs. While in the validation architecture, every transmitter of the U2U link adopts the action that can maximize the evaluation Q-value, based on the well-trained target network at every time step. Accordingly, the results of one episode were recorded and are shown in the simulation  section.
**Algorithm 1** The improved DQN for multi-UAV formation resource optimization with the CBAM and VDN (training stage)Initialize replay memory buffer to capacity *D*.Initialize the Q-network of the UAVs with random parameters θ and a target network of UAVs with parameters $\hat{\theta }=\theta$.Activate environmental simulator, generating the locations of all the UAVs. Initialize the action space *A*, including the power and frequency each UAV can choose.**for** step=1, *M* **do**   Update positions of UAVs, channel information of U2B links $H_{UB}$, the U2U links $H_{uu}$, the interference from U2U links $I_{uu}$, the uplink reuse indicator of U2U links *B*, the time left to transmit data $E_{t}$ and the data left to transmit.   **for** episode =1, *T* **do**     The U2U link selects a random action with probability ε, otherwise, select $a_{t}=\max_{a_{t}\in A}Q\left(s_{t},a;\theta \right)$ with probability 1−ε.     Implement $a_{t}$ and move on to the next state $s_{t+1}$ with a reward $r_{t}$. Store the transition $\left(s_{t},a_{t},r_{t},s_{t+1}\right)$ into the memory buffer.     A certain amount of mini-batch transitions $\left(s_{t},a_{t},r_{t},s_{t+1}\right)$ is sampled randomly from the memory buffer. Preprocess the collected state data and input the processed data into the first convolution layer of the Q-network and output the data $M_{cnn}$.     1. Input data $M_{cnn}$ into the CBAM module to obtain the data $M_{CBAM}$.     The q-values are obtained after the following convolution and relu operations are carried out on the output of the last layer.     2. Perform VDN operations to obtain the summation reward Q.     Set $y_{t}=\left\{\begin{array}{c} r_{j},\;\;\;\;\;\;\;\;\;\;\;\;\;\;\;\;\;\;\mathrm{if}\;\mathrm{ends}\;\mathrm{at}\;\;\mathrm{step}\;t+1\\ r_{t}+\gamma \max_{a_{t}}Q\left(s_{t+1},a_{t};\hat{\theta }\right),\mathrm{otherwise} \end{array}\right.$.     Perform a gradient descent policy on the loss function ${\left(y_{j}^{tot}-Q\left(\tau_{j},u_{j},s_{j};\theta \right)\right)}^{2}$ to improve the coefficients θ.     Every *K* step, let $\hat{\theta }=\theta$.   **end for****end for**Save the coefficients of the target network.

## 4. Simulation

The selection of the simulation parameters are based on existing works [18] and 3GPP specifications [34]. Multiple UAVs were arranged as a cube formation in a three-dimensional 2 km × 2 km × 2 km area. The main parameters are shown in Table 1. In the training stage, the size of the input and output CNN layer convolution kernel was 3 with a padding of 1. The global average-pool and max-pool operator in the CBAM module was 6 ∗ 6 and the channel ratio r was 16. To verify the generalization of the proposed method, scenarios with different numbers of UAVs were tested. To prove the validity of the proposed method, first the experiment with only a traditional DQN was executed, then, the CBAM and VDN were embedded separately and lastly both the CBAM and VDN were embedded simultaneously. The experimental results were recorded, analyzed and compared to the random and multichannel access methods proposed in [17] and [27], respectively. For the random method, the uplinks and power were randomly allocated to the UAVs. For the multichannel access method, the power used by every UAV was not changed, and the accumulated discounted reward was maximized to realize the optimization of the channel allocation.

**Figure 2:**
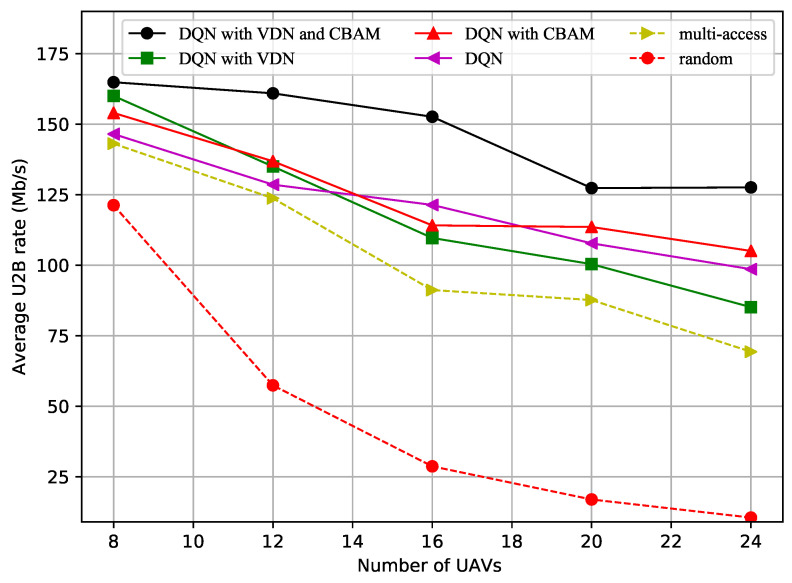
Average U2B rate vs. number of UAVs.

Figure 2 shows how the average U2B link data transfer rate varied with the number of UAVs. More interference was generated as the number of UAVs increased, which caused the decrease in the average data transfer rate. The average data transfer rate, obtained using the random and multichannel access methods, was always lower than that obtained by other methods, especially the DQN with CBAM or with both the VDN and CBAM. From this it can be inferred that the improved DQN with CBAM method, or the method with both the VDN and CBAM, will maintain a high communication rate as more UAVs are added to the multi-UAV formation.

**Figure 3:**
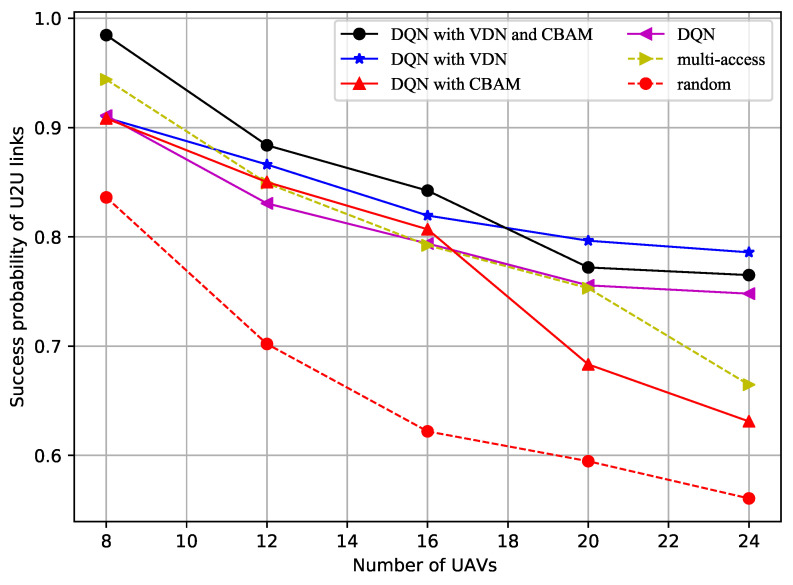
Success probability of the U2B links vs. number of UAVs.

Figure 3 shows how the successful data transfer probability of the U2U links varied with the number of UAVs. More interference was generated as the number of UAVs increased, which caused the decrease in the successful data transfer probability. The successful data transfer probability obtained using the random method was always the lowest. When there were only 8 UAVs, the successful data transfer probability using the multichannel access method was not low, but it went down quite fast as the number of UAVs increases. From this figure, it can be inferred that the improved DQN with VDN method, or the method with both the VDN and CBAM, will maintain a high successful data transfer probability, as more UAVs are added to the multi-UAV formation.

**Figure 4:**
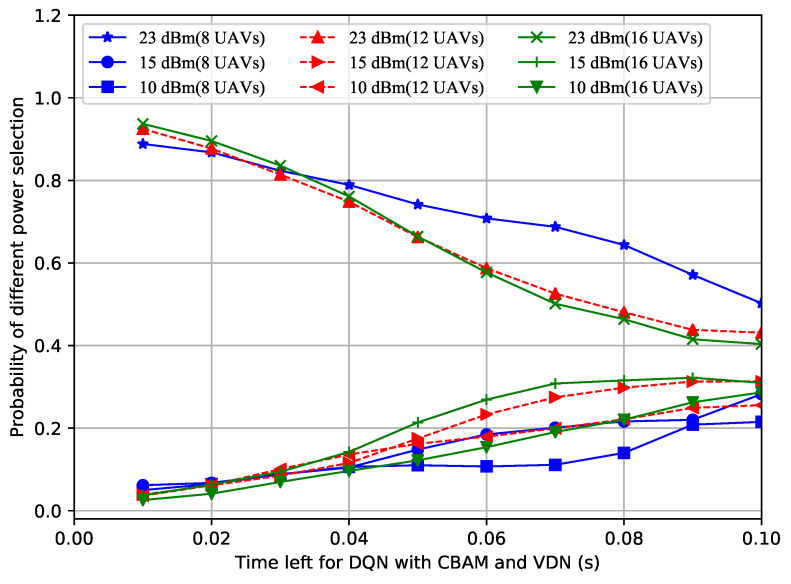
Probability of power selection vs. remaining time for the DQN with the CBAM and VDN.

**Figure 5:**
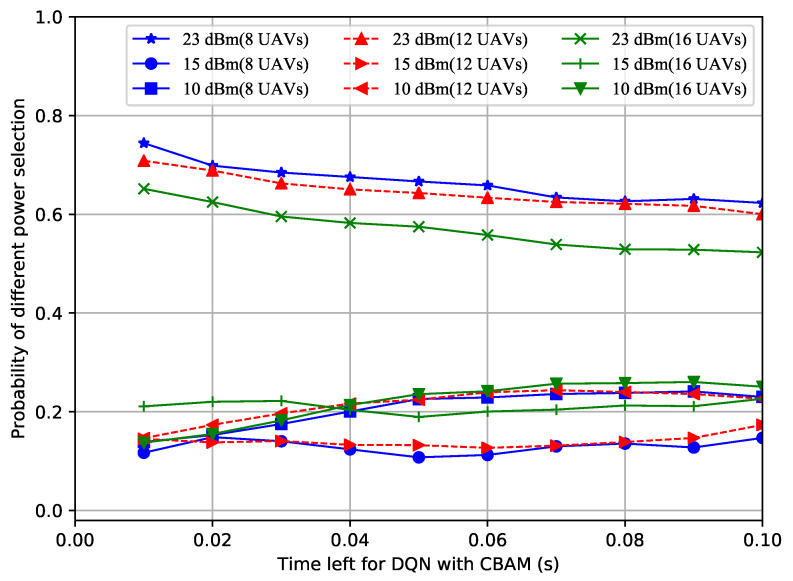
Probability of power selection vs. remaining time for the DQN with CBAM.

**Figure 6:**
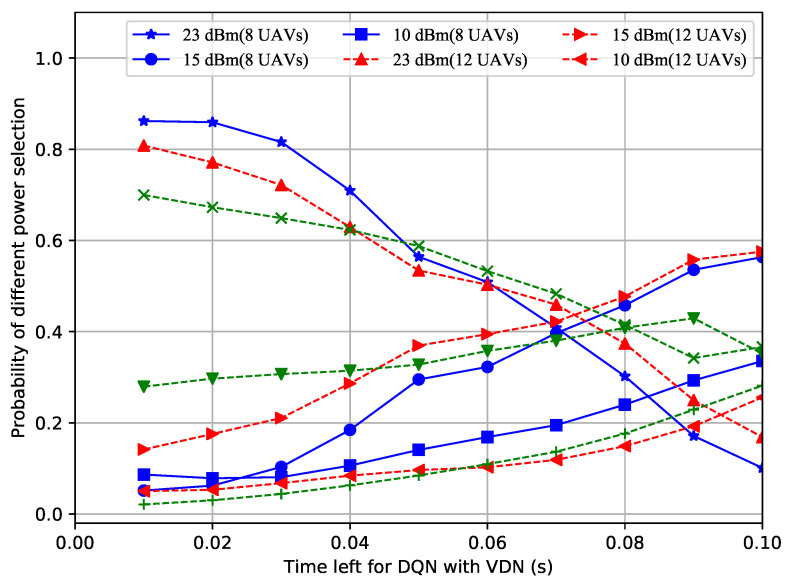
Probability of power selection vs. remaining time for the DQN with VDN.

In order to figure out why the proposed improved DQN mechanisms showed superior performances, the power selection situations of the different methods with a different number of UAVs over time were recorded. Shown in Figure 4, Figure 5 and Figure 6, the probability of the power selection with 8, 12 and 16 UAVs for three situations was drawn, respectively. By observing these three figures, it can be inferred that because the UAVs can wisely select different powers, less interference is generated in the system. In addition, when the number of UAVs in the formation varies, the UAVs automatically adjust the number of lowest or highest power users.

**Figure 7:**
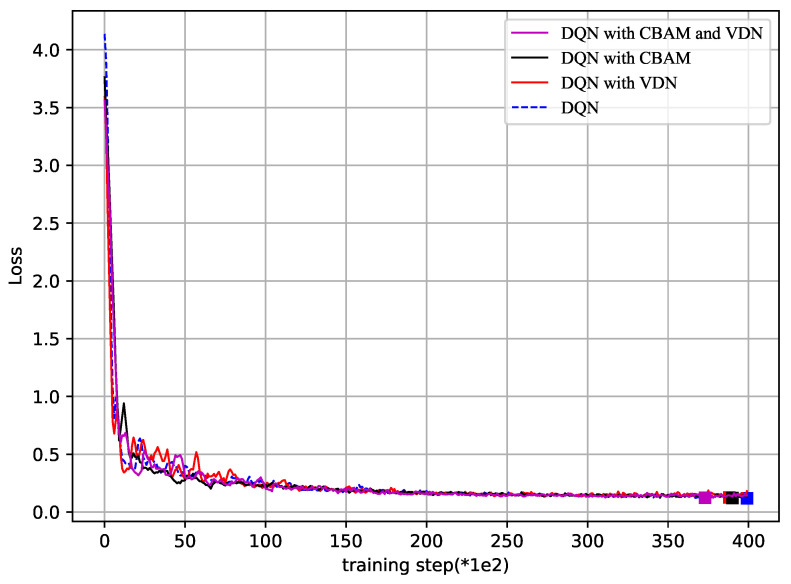
Loss vs. training step (*1e2).

**Figure 8:**
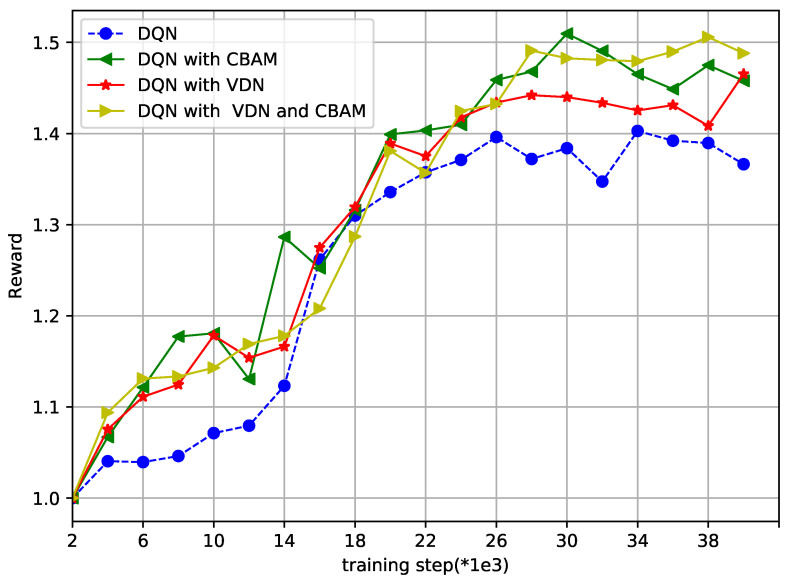
Reward vs. training step (*1e3).

Finally, Figure 7 and Figure 8 show how the reward and loss varied in the overtraining step, respectively, to show the convergence of the proposed DQN and the improved DQN. The axis label “training step()” represents every number times 100 or 1000, with a maximum of 40,000 training steps. With 40,000 training steps, the reward and loss gradually converged, despite some fluctuations. From Figure 7, the reward was bigger in the improved DQN than that in the DQN method. In Figure 8, the lowest points of loss are marked. It is obvious that the DQN with the VDN or CBAM always reached the lowest point earlier than with the DQN alone, and the method with both the VDN and CBAM was the earliest one. The two figures further illustrate the effectiveness and convergence of the improved method.

## 5. Conclusions

- This manuscript focuses on the realization of joint power and spectrum resource optimization using a DQN mechanism for a multi-UAV formation communication network, in which the UAVs are located in 3D forms when the multi-UAVs are on a mission. The objective was to maximize the transmission rate and increase the successful data transfer probability simultaneously.
- The main idea was that the U2U links were treated as agents and the CBAM and VDN were further introduced based on the DQN. Compared to the random and multichannel access methods, the DQN method slightly improved system performance, and the introduction of the CBAM and VDN further improved the data transfer rate of the U2B links and the successful data transfer probability of the U2B links.
- The reason for the performance improvement is that the UAVs could intelligently choose the appropriate power and frequency based on the remaining time and the number of UAVs. The authors drew the conclusion that the superiority of the proposed method became more and more obvious as the number of UAVs in the formation increased.
- This model can be used in agricultural or military applications, such as disaster relief, environmental detection, remote situational awareness, deception and jamming. Even if a few UAVs break down, the overall capability of the UAVs will not be affected. However, the speed is low and the mobility and self-defense ability is poor. If the number of UAVs is small, they are easy to detect and intercept, and the different flight altitudes will affect the communication quality between the UAVs and the ground base station. In this work, the formation of the UAVs remained the same during the execution of tasks, and future work could focus on the situation with changing formations.

## Figures and Tables

**Table 1:** Simulation parameters.

Parameters	Value
Number of UAVs (training)	8
Number of uplink	8
Number of UAVs (testing)	8/12/16/20/24
Carrier frequency	1 GHz
Noise variance	−96 dBm
U2U link transmitter power	23 dBm/15 dBm/10 dBm
U2B link transmitter power	23 dBm
U2B channel parameter ηNLoS	20
U2B channel parameter ηLoS	1
U2B channel parameter ω	2
U2B channel parameter β	0.135
U2B channel parameter α	12
U2U channel power gains factor *h*	−31.5 dB

## Abbreviations

The following abbreviations are used in this manuscript:

DQN	deep Q-network;
UAVs	unmanned aerial vehicles;
U2B	UAV-to-base station;
U2U	UAV-to-UAV;
CBAM	convolutional block attention module;
VDN	value decomposition network;
DL	deep learning;
RL	reinforcement learning;
ML	machine learning;
DRL	deep reinforcement learning;
CNN	convolutional neural network;
BS	base station;
LoS	line-of-sight;
AWGN	additive white Gaussian noise;
NLoS	non-line-of-sight;
ITU	International Telecommunication Union;
SINR	signal-to-interference-plus-noise ratio;
CMDP	constrained Markov decision process;
NN	neural network;
ATG	air-to-ground.
